# Continual Cell Deformation Induced via Attachment to Oriented Fibers Enhances Fibroblast Cell Migration

**DOI:** 10.1371/journal.pone.0119094

**Published:** 2015-03-16

**Authors:** Sisi Qin, Vincent Ricotta, Marcia Simon, Richard A. F. Clark, Miriam H. Rafailovich

**Affiliations:** 1 Materials Sciences and Engineering Department, Stony Brook University, Stony Brook, NY, United States of America; 2 Dental School, Stony Brook University, Stony Brook, NY, United States of America; 3 Department of Bioengineering, Stony Brook University, Stony Brook, NY, United States of America; Pennsylvania State Hershey College of Medicine, UNITED STATES

## Abstract

Fibroblast migration is critical to the wound healing process. In vivo, migration occurs on fibrillar substrates, and previous observations have shown that a significant time lag exists before the onset of granulation tissue. We therefore conducted a series of experiments to understand the impact of both fibrillar morphology and migration time. Substrate topography was first shown to have a profound influence. Fibroblasts preferentially attach to fibrillar surfaces, and orient their cytoplasm for maximal contact with the fiber edge. In the case of en-mass cell migration out of an agarose droplet, fibroblasts on flat surfaces emerged with an enhanced velocity, v = 52μm/h, that decreases to the single cell value, v = 28μm/h within 24 hours and remained constant for at least four days. Fibroblasts emerging on fibrillar surfaces emerged with the single cell velocity, which remained constant for the first 24 hours and then increased reaching a plateau with more than twice the initial velocity within the next three days. The focal adhesions were distributed uniformly in cells on flat surfaces, while on the fibrillar surface they were clustered along the cell periphery. Furthermore, the number of focal adhesions for the cells on the flat surfaces remained constant, while it decreased on the fibrillar surface during the next three days. The deformation of the cell nuclei was found to be 50% larger on the fiber surfaces for the first 24 hours. While the mean deformation remained constant on the flat surface, it increased for the next three days by 24% in cells on fibers. On the fourth day, large actin/myosin fibers formed in cells on fibrillar surfaces only and coincided with a change from the standard migration mechanism involving extension of lamellipodia, and retraction of the rear, to one involving strong contractions oriented along the fibers and centered about the nucleus.

## Introduction

It has been nearly 20 years since Grinnell et al [1–4] first proposed that cell migration studies be performed in a 3-D collagen environment which mimics the human skin ECM. The ECM is a very complex system of fibers composed of a variety of different proteins such as collagen and fibronectin, whose sizes range from nanometer to micrometer. Cell migration, a critical process in wound healing, [5, 6] has been shown by numerous groups to be a function of substrate topography [7–12]. The micro-droplet technique is an accepted method for measuring cell migration, simulating wound healing, and allowing for the study of chemotaxis and haptotaxis. Yet, most studies, utilizing this method were performed on flat surfaces. In the case of fibroblasts, the “sunburst” or patterns of rays emanating from a central source, observed were shown to result from haptotaxis as the cells try to maximize the distance between adjacent cells. Liu et al [13] compared the migration of cells on flat surfaces to that on fibrous mats and found some fundamental differences. Measuring the migration velocity as a function of distance from the droplet, over a period of 24 hours, they found that on flat surfaces, the cells move fastest as they exit the droplet, but slow down as the distance between them increases, reaching a terminal velocity similar to the single cell value. When the droplets were placed on a mat of parallel fibers with diameters greater than 8 microns, the cells organized to form a ring around the perimeter of the droplet, and exited by moving only along the fibers. Therefore, for the first 24 hours, the distance between cells remained constant with time, being determined by the fiber pattern rather than the cell trajectory. The cell velocity also remained constant at the single cell value, which was much lower than the exit velocity on the flat film.

McClain et al studied the time scale for healing of punch wounds in a Yorkshire pig model and found a three day lag period before the onset of granulation tissue formation [14]. Since granulation tissue forms via en mass fibroblast cell migration, we wanted to investigate the nature of the cell velocity on different substrates after the first 24 hours. Even though the in-vivo process is more complex, being the result of multiple factors, here we focused on the influence of substrate morphology by measuring the migration for up to seven days and correlating the results with changes in cell and nuclear morphology, cell metabolism, and expression of vinculin and myosin IIA.

## Materials and Methods

### Fabrication of PMMA thin film and microfibers

Clean glass coverslips were coated with a thin film of PMMA (Mw = 120,000 Da, Mw/Mn = 3; Sigma-Aldrich inc., St Louis, MO) which were spun cast from toluene solution ((Fisher Scientific, Pittsburgh, PA) at a concentration of 30mg/mL by at 2500PRM for 30 seconds. Samples were then annealed at 120°C in a vacuum of 10^−7^ Torr overnight to remove the remaining solvent, remove stress in the film, and sterilize the substrates. Fiber scaffolds were generated by electrospinning different PMMA solution and colleting by a rotating drum at 6750 r/min. Before seeding the cells, all samples had been sterilized under ultraviolet (UV) light for 20 min. Then, a solution of 30μg/ml intact human plasma fibronectin (Fn) (Millipore, Temecula, CA) solution in serum free Dulbecco’s Modified Eagle medium (DMEM) was added at 37°C for 2 h. Similar procedure was used for the coating of collagen (PureCol, San Diego, CA)

### Cell culture and cell migration assay

Adult Human Dermal Fibroblasts (CF29) were purchased from ATCC, and the experiment only used cell passages from ten to twelve. Cells had been routinely cultured in Dulbecco’s Modified Eagle medium (DMEM), with 10% fetal bovine serum (Hyclone, Logan, UT) and antibiotic mix of penicillin, treptomycin, and L-glutamine (GIBCO BRL/Life Technologies, Grand Island, NY) in a humidified incubator at 37°C. The traditional agarose gel migration assessment was performed. The membranes of fibroblasts were stained with DiD dye, and then re-suspended in a volume of 0.2% (w/v) agarose solution to obtain the final cell density of 1.5×10^7^ cells/ml. The agarose droplet was then introduced to the sterilized sample with micropipette, and each droplet was of 1.25μL. Samples with cells were then placed at 4°C for 10 min to allow the agarose to solidify. After cooling, full- DMEM was added to each sample. Cells were then cultured in the 37°C incubator with 5% CO_2_ for 4 days.

### Measurement of cell migration speed

Time-lapse images of fibroblasts cell migration are recorded by MetaMorph-operated CoolSNAP HQ camera (Universal Imaging Corporation, Downingtown, PA) attached to a Nikon Diaphot-TMD inverted microscope fitted with a 37°C incubator stage and a 10× objective lens. Images were automatically taken each 15 min and for total 60 min, so 5 pictures were taken every time. Migration velocity could be calculated by measuring the total migration distance divided by migration time. Single cells that were at the leading edge of migration were chosen because they there are no cell-cell interference. Dividing cells and those that were out of focus plane are excluded. Final data was calculated after repeating the above experiment for at least 4 times and measuring at least 20 cells, with 3 replicates.

### Immunofluoresent staining

Cells were rinsed with PBS, fixed with 3.7% formaldehyde for 20 min, then permeabilized with 0.4% Triton for 7 min, and blocked with 2% BSA in PBS for 30 min at room temperature. Focal adhesions were visualized by immunostaining for vinculin (Sigma, Saint Louis, MO), at a 1:600 dilution for 1 h then incubated with the Oregan Green 488 goat anti-mouse secondary antibody (Invitrogen, Carlsbad, CA) at a 1:600 dilution for 1 h at room temperature. Similar method was used for the myosin IIA (Cell Signaling Technology, Danvers, MA) staining. Nuclei were stained with 4’,6-diamiadino-2-phenylindole (DAPI, Sigma-Aldrich, Inc., St. Louis, USA) for 10 min at room temperature. F-actin was stained with phalloidin (Invitrogen, Carlsbad, CA) for 20 min. Samples were then imaged by a Leica TCS SP2 laser scanning confocal microscope (Leica Microsystems, Bannockburn, IL) with water objective lens. The number of vinculin-positive focal adhesion sites, the aspect ratio of nuclear, and the intensity of mysion IIA staining were quantified by Image J.

### XTT assay for cell metabolism

The standard XTT assay kit was purchased from Roche (Indianapolis, IN), and followed the company procedure. The initial cell density was 2500cells/well with 400 μL medium. After 4 days incubation, a mixture with 50:1 ratio of labeling reagent and electron-coupling reagent was added to the medium and detected by an Bio-RAD microplate reader (Hercules, CA) at 450 nm after 4 hrs in a humidified incubator.

### RT-PCR of myosin IIA

Myosin IIA mRNA levels were determined using quantitative real time polymerase chain reaction (qRT-PCR) of cDNA. In order to ensure that all cells analyzed experienced the same surface environments, rather than having to emerge from a droplet, the cells were plated directly on either FN coated PMMA fibers or flat PMMA spun cast films. As will be discussed later, the cells experienced the same speed on the surfaces whether they exited from droplets, or were plated directly. Samples were first washed twice with PBS, detached from each surface with trypsin (0.05%; Gibco Trypsin) and harvested by centrifugation. The cell pellet was then lysed and RNA isolated with the Qiagen RNEasy Kit according to the manufacturer’s instructions (RNeasy kit, Qiagen, Valencia, CA). To prepare cDNA, 1 μg of total RNA was reverse transcribed with Superscript II Reverse Transcriptase (200 units/reaction; Invitrogen). The cDNA was then used as a template for qRT-PCR with primers for myosin IIA (forward: TACGCTGAGGAACACGAACC and reverse: TCCCGTCCATGAACCCTTG) and for 18S RNA (forward: GTAACCCGTTGAACCCCATT and reverse: CCATCCAATCGGTAGTAGCG) which served as control for normalization. qRT-PCR was performed at the Stony Brook University DNA Sequencing Facility using the SYBR Green PCR Kit (Qiagen, Valencia, CA) and controlled in a DNA Engine MJ Opticon 2 Thermal Cycler with continuous fluorescence detection (MJ Research Inc., Union, NJ). Following a 15 min incubation at 95°C, amplifications were performed with 40 cycles of 94°C (30 sec), 55°C (30sec) and 72°C (30 sec). A melting curve program was performed immediately after the above cycling program in order to generate a first derivative dissociation curve. Each sample was assessed in triplicate.

### Statistics

Statistical analysis of the data was performed using GraphPad Prism (version 6) with an unpaired t test and Welch’s correction. The typical sample size used exceeded n = 30 for each data set. The p values for each set of data groups is presented on the graph by the symbol, *, which represents P<0.001. Values of P>0.05 were not considered significant.

## Results

### Migration Speed

**Fig 1:**
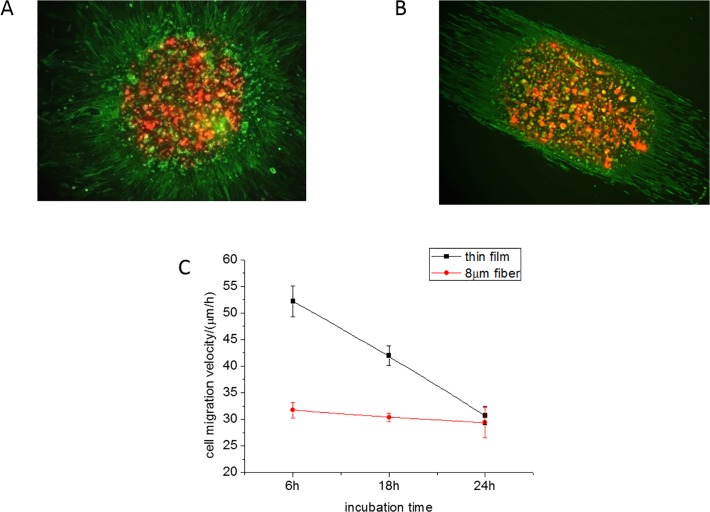
En-mass cell migration within 24 hours. The overlapped image of en-mass cell migration of live cells stained with DiD (red), and incubated for four hours, onto the image of cells incubated for 24h, fixed and stained for F-actin with Alexa Fluor (green) (a) On a spun cast, FN coated PMMA thin film and (b) On electrospun FN coated, PMMA microfibers. The lines are drawn to guide the eye towards the perimeter of the migration front at 4 and 24 hours, respectively. Error Bar = 250 μm. (c) The en-mass cell migration velocity, as measured from the motion of the front, on the thin film (black) and 8μm fibers (red) as a function of time.

In Fig. 1A, 1B we show fluorescent microscope images of the droplet and the cells emanating from the droplet onto spun cast flat PMMA film substrates and parallel electrospun PMMA fibers, both coated with fibronectin. The images are formed by a superposition of the image taken after four hours (red, DiD), onto the image of cells obtained after incubation for an additional 24 hours, after which the cells are fixed and stained for F-actin with Alexa Fluor (Green). From the figures we can see that immediately upon emergence, the pattern of the migration differs between the cells on the flat film and the cells placed on the fibers. Since the chemical composition of the two types of substrates is the same, the differences reflect primarily substrate topography and are a consequence of the confinement of the cells. The cells on the flat film form a sunburst pattern as they emanate from the droplet (Fig. 1A), where their trajectories reflect a tendency of fibroblasts to migrate away from each other. In contrast, those coming out from the droplet placed on the fibers (Fig. 1B), orient themselves along the fibers and migrate only in the fiber direction. The velocity, as measured from the distance traveled from the droplet edge to the perimeter of the migrating cells, is plotted as a function of time in Fig. 1C. From the figure we find that the cells emerge from the droplet with an initial velocity which is approximately 60% greater on the flat than on the fibrillar surface. With increasing time, the distance between cells increases and the migration velocity decreases, approaching the single cell value after 24 hours. In contrast, the cells placed on the fibrillar substrate emerge from the droplet with the single cell migration velocity, which remains constant for the first 24 hours. These results are consistent with those previously reported study [13] where they explained their results in terms of a haptotaxis like phenomenon due to cell crowding, as opposed to a chemotaxix effect where soluble factors were present. Fibroblasts put out cell processes which can sense adjacent cells. On the flat surface, the cells chose a trajectory which would continuously increase the distance between cells. The largest speed was observed at the smallest cell-cell distance. On micron sized fibers, once the cells emerged from the droplet, the cell-cell distance was determined by the fibers which on the oriented fiber surface remained constant with minimal cell-cell contact. Hence in the absence of a haptotaxic gradient, the cell migration speed remained constant at the value of the single isolated cell for the first 24 hours.

**Fig 2:**
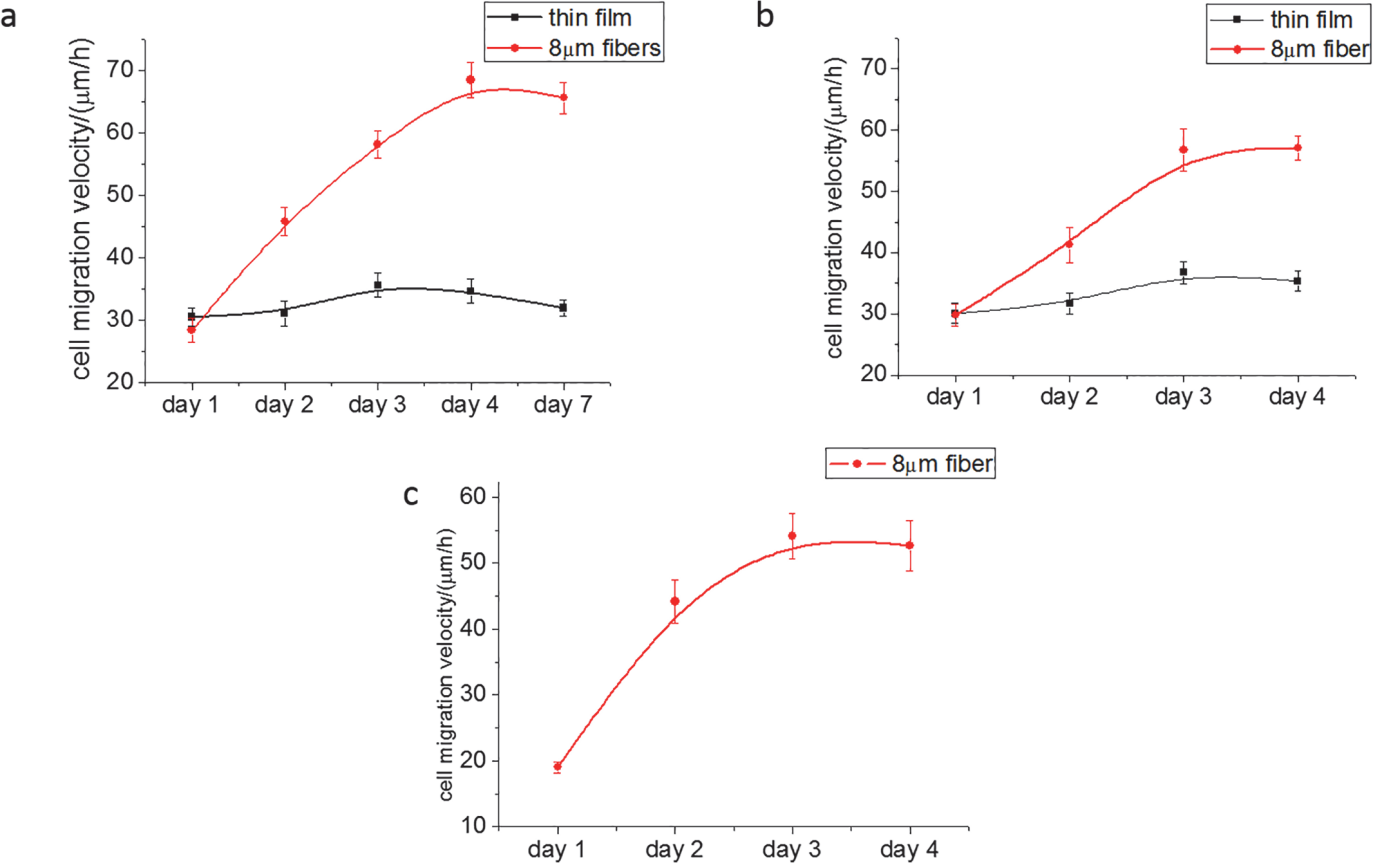
Cell migration velocity after 24 hours under different conditions. (a) En-mass cell migration velocity on FN coated thin film and 8μm fibers. (b) Single cell migration velocity on FN coated thin film and 8μm fibers for 4 days. (c) En-mass cell migration velocity on collagen coated 8μm fibers for 4 days.

In Fig. 2A we plot the migration speed, measured at 24 hour intervals, during the subsequent three days following the initial 24 hours. From the figure we see a dramatic reversal of the response. The cells migrating on the flat films are now moving at the constant single cell velocity, while those in the fibrillar surfaces are accelerating at a constant rate of |a| = 0.56μm/h^2^, reaching, by the fourth day, a velocity nearly double the single cell value of v = 68.5 μm/h, and exceeding the initial velocity of those on the flat surfaces. Further measurements from day 4 to day 7, show that no further change in the velocity on both flat and micron fiber substrates.

In order to rule out any chemotaxic or haptotaxic effects from the droplet, we also measured the single cell migration speed with incubation time, where cells were plated directly on the substrate at an initial cell density of 2500cells/well without the agarose droplet. The results are shown in Fig. 2B, where we find that on the flat surfaces the magnitude of migration speed after 24 hours is the same as which achieved by the cells that had migrated out of the droplet, confirming that the cells migrating on the perimeter of the droplet had achieved single cell behavior. The response of the cells to increasing incubation time is also similar to that observed when the cells are initially plated within the droplet, namely the migration speed of the cells plated on the flat films remains constant for the four day observation period, while the speed of the cells plated on the fibrillar surface increases with the same constant rate of |a| = 0.56 μm/h^2^. The only difference between the cells plated directly on the substrate and those emanating from the droplet, is the magnitude of the plateau speed = 59.1μm/h which is reached at day 3, rather than day 4, and is similar to the value 52.2μm/h of the cells existing the droplet on the flat surface. Hence the increase in migration speed appears to be caused by the fibrillar topography of the substrate rather than a consequence of the en-mass behavior of the migration imposed by the crowded condition in the droplet.

In order to rule out any effects specific to fibronectin, the fibers were also coated with collagen and the en mass velocity of the cells emanating from a droplet was measured as a function of time. From the results plotted in Fig. 2C, where we can see that even though the initial velocity is somewhat lower, the functional behavior is similar to the single cell response shown in Fig. 2B. The migration velocity increases linearly, reaching a plateau at v = 60.0μm/h on the third day, and hence the phenomenon is not related to activation via any functional domains specific to fibronectin.

### Morphology and metabolic activity

**Fig 3:**
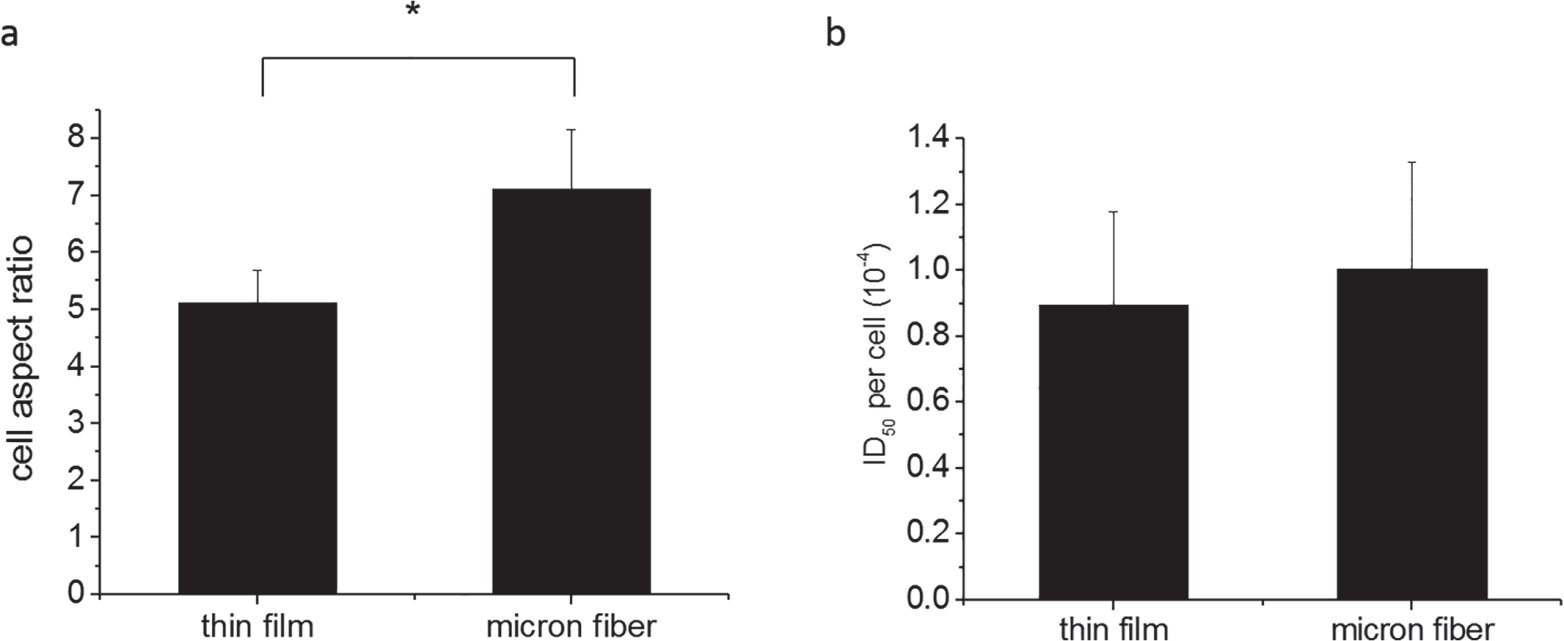
Cell aspect ratio and ID_50_ per cell reading on thin film and microfibers at day 4. (a) Cell aspect ratio was calculated as: the length of the cell/width of the cell plated on thin film and micron fiber surfaces, P<0.001 (*). (b) The XTT assay at day 4 was performed and followed with a DAPI nuclear staining. The reading of ID_50_ value reflects the metabolism level of all the cells at day 4. However, the cell proliferation varied on different surfaces. As a result, we divided the ID_50_ value by the actual nuclear number counted after DAPI staining, and evaluate the metabolism level per cell. P>0.05 which was not considered as significant.

Observation of the morphology of the cells plated on the flat and fiber surfaces shows that the cells on the flat surface have multiple orientations, which are continuously changing during the migration process, while those on the fibers remain orientated along the fiber direction. In Fig. 3A we plot the time averaged aspect ratio of the cells at day 4 on the flat and fibrillar surfaces, where we see that the value on the fibrillar surfaces is significantly larger, 7.1 vs 5.1 (p<0.001).

In order to determine whether placement of the cells on the fibers affects the rate of metabolism, an XTT assay, was performed on day 4, and as shown in Fig. 3B, no significant differences were observed between the cells plated either on the flat surfaces versus the electrospun fiber surfaces. Hence no additional mitochondrial activity occurs as a result of the cell continuously conforming to the three dimensional topography of the fibers.

### Localization of vinculin

**Fig 4:**
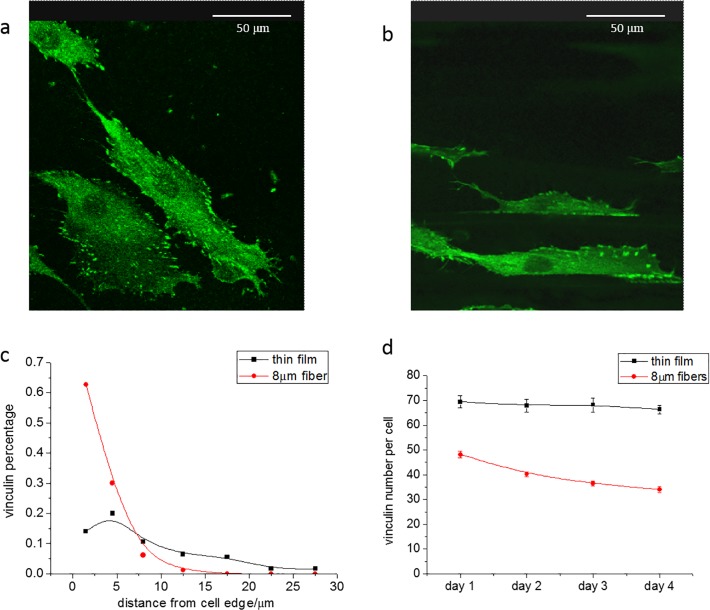
Vinculin distribution on flat film and 8μm fibers. (a) Confocal images of vinculin distribution on thin film at day 4 (b) Vinculin distribution on 8μm fibers at day 4. (c) The distribution of vinculin as a function of distance from cell boundary at day 4. The percentage of vinculin number was plotted as a function of the distance between vinculin locations to the edge of the cell. (d) The number of vinculin per cell on different surface for 4 days.

The migration velocity is closely related to the number and size of focal adhesion sites for the cells on the substrate [11, 15, 16]. To achieve the maximum velocity, cells must be able to form optimal strength of focal adhesions. If the adhesion is too strong cells will migrate at a low speed, and if the adhesion is too weak, cells will not be able to exert adequate traction forces and will be unable to migrate. A recent study has shown that the size of the focal adhesions may be correlated to the cell migration [17]. We therefore, stained the cells using fluorescent secondaries against anti-vinculin, obtained images with confocal microscopy, and the results are shown on Fig. 4A, and 4B. From the figures, we can immediately see that the pattern of focal adhesion contacts, as determined from the vinculin stain, varies drastically between the cells plated on the flat films and those plated on the 8μm fibers. On the flat films one can see focal adhesions distributed both at the periphery of the cells as well as on the interior, whereas on the 8μm fibers the focal adhesions were clustered only along the edges, following the contours of the fibers. The distribution of the focal adhesion points can be quantified by plotting the percentage of the focal adhesions present at different distances from the cell edge. The results are shown in Fig. 4C, where we can see that on the flat surface only approximately 20% are within 5μm of the cell edge, with the remainder distributed almost uniformly up to 35μm from the edge. On the fibers more than 60% are within 5μm, with a sharp decrease to only a few percent at 10μm from the edge. The distribution of loci of the focal adhesions may reflect the mechanism of motion of the cells. On the flat surfaces the cells migrate either radially outward from the droplet or along random directions, following processes which may be extend in all directions. On the fibers though, motion occurs in only along the fiber, and the cell shape is defined by the edge of the fiber.

The number of focal adhesion points per cells was also counted and the results are plotted in Fig. 4D as function of incubation time. From the figure we see that the number of focal adhesion per cell on day one is 70 on the flat films vs. only 50 on the 8μm fibers, even though at this time the migration velocities on the two substrates are nearly the same. With increasing incubation time the number on the flat films remains constant while the number on the 8μm fibers decreases gradually to 35 by the fourth day. The decrease in focal adhesion number is consistent with the increase in migration speed during the same period of time on the 8μm fibers. The lack of change in number on the flat surface is consistent with the lack of change in the speed of migration on these substrates.

### Aspect ratio of the nucleus

In the pervious study, we have shown that on flat surfaces, migration was triggered by nuclear deformation [18], which initiated a cycle of traction force exertion to reduce deformation and ultimately resulted in center of mass translocation. Conversely, it was recently shown that inhibition of nuclear deformation via physical constraints led to complete cessation of cell migration [19]. We therefore investigated whether nuclear deformation may also be involved in triggering the enhanced migration on the fibrillar surfaces.

**Fig 5:**
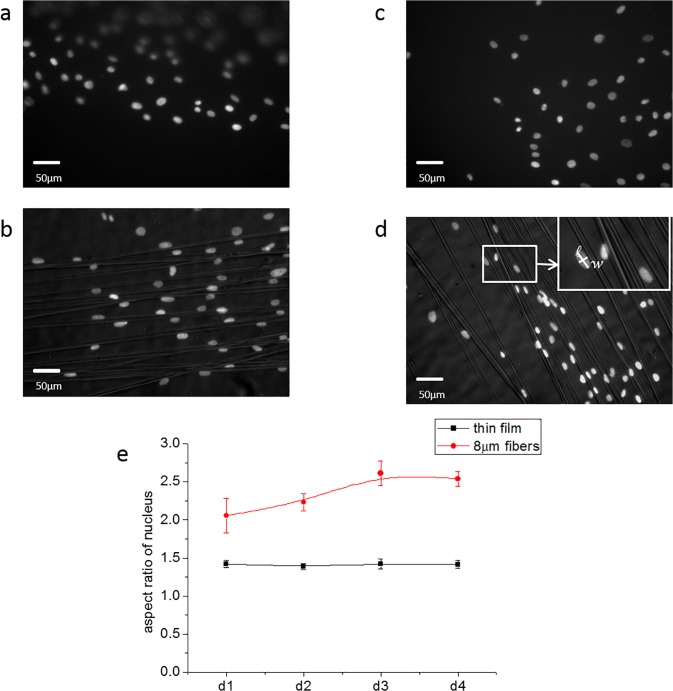
Nuclei morphology on different surfaces. The nuclei morphology at day 1 on (a) thin film (b) 8μm fibers, (c) Nuclei morphology at day 4 on thin film (d) 8μm fibers. (e) Measurement of cell aspect ratio on different surfaces for 4 days.

In order to observe the nuclear structure we stained the nucleus of cells cultured on the different substrates with DAPI, and their aspect ratio was measured (Fig. 5A-5D). From Fig. 5E, we found that the nuclei of the cells on the flat films ranged from spherical to slightly (Rmax~2.1) elongated, with the mean elongation R~1.4 remaining constant over the four day observation period. The relatively large dispersion reflects the fluctuation in nuclear aspect ratio previously reported by Pan et al to be associated with different stages of the migration process on flat surfaces. On the fibrillar surfaces the aspect ratio was larger immediately after the cells exited from the droplet, with an average value of R = 2.0 after 24 hours. This value increased gradually reaching a plateau of R = 2.5 on the third day. Since nuclear deformation is a vital part in cell migration, [20–22] it is tempting to postulate that it may be responsible for the acceleration of the cells on the fibers, where nuclear deformation is observed to increase during the same period as the cell migration velocity increases. In contrast, on the flat surfaces, both the mean velocity and the mean nuclear deformation remain constant over this period of time.

### Intensity of myosin IIA and cell contraction

**Fig 6:**
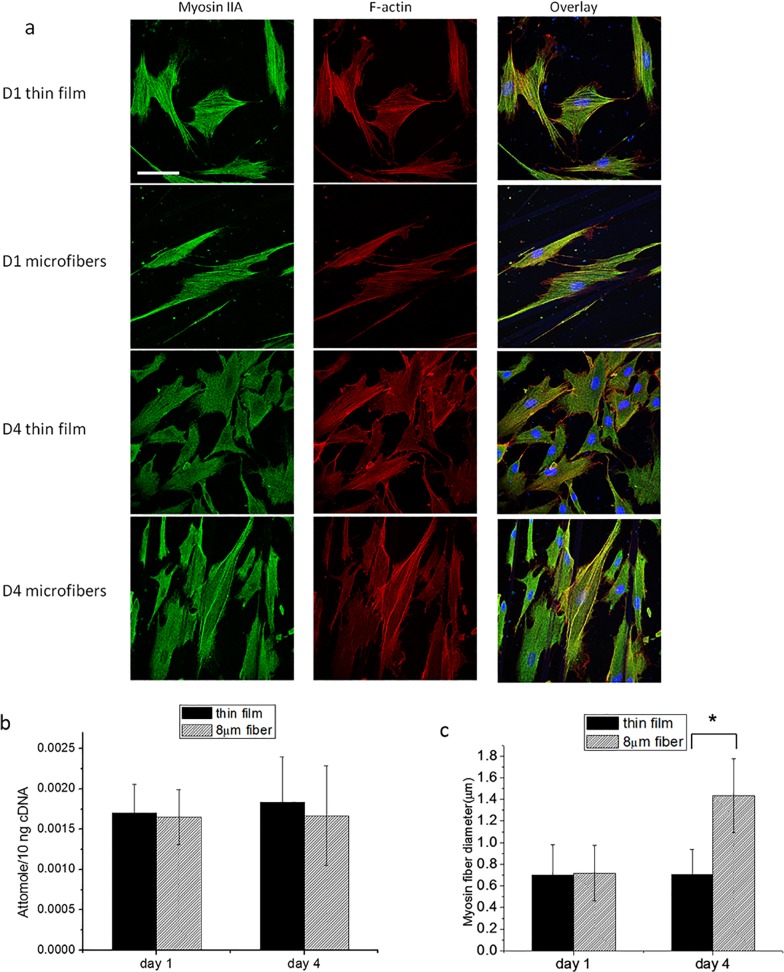
Immunofluorescent staining, RNA expression, and fiber diameter of Myosin IIA at day 1 and day 4. (a) Confocal images of cells stained with myosin IIA (green), F-actin (red), and the merged pictures on thin film and 8μm fibers. Error bar = 75μm. (b) RT-PCR result of MyosinIIA (2208F-2440R) expression on thin film and 8μm fibers P>0.05: not significant. (c) Myosin fiber diameter on thin film and 8μm fibers *: P<0.001.

Myosin IIA is another important protein associated with cell migration since it plays a large role in transmitting the traction forces responsible for cell contraction as well as nuclear deformation [23, 24] during migration. Traction forces are transmitted via fiber bundles consisting of actin/myosin complexes where the actin fibers are contracted via connections to the myosin, hence transmitting forces due to focal adhesions with the surface [25–29]. In addition Myosin II also regulates vinculin recruitment and focal adhesions, which in turn determine migration velocity [30]. In order to observe Myosin fiber formation we stained the cells with immunofluorescent antibodies against Myosin IIA. We chose Myosin IIA over Myosin IIB since it is more prominent [31] in generating forces in nonmuscle cells. The results are shown in Fig. 6A for cells on the 8μm fibers and on flat substrates. In all images we found uniform myosin staining across the cell cytoplasm, where the intensity does not vary significantly between cells plated on flat or fiber surfaces that incubated for one or four days. This finding is consistent with the RT-PCR data (Fig. 6B) where no significant differences were detected in the levels of Myosin IIA RNA.

Closer examination of the images (expanded segment of typical cells) show that on day one the myosin is organized into thin fibrils, approximately 0.7μm in diameter, which are extended across the long axis of the cell. These fibrils appear on all samples but are more pronounced on the flat surfaces. In Fig. 6A we show the corresponding images for the cells, which also stained for F-actin. Comparing the figures, we can see that the distribution of the thin myosin fibers parallels that of the actin fibers which is consistent with the formation of the actin/myosin complexes reviewed in the literature [24, 32].

The appearance of the cells on the flat substrates does not change much between days 1 and 4. On the other hand, on the fibrillar surfaces, much thicker fibers become visible which span the length of the cells, and are oriented parallel to the underlying direction of the 8μm fibers. Even though these fibers appear brighter, the intensity ratio of actin to myosin is not significantly different from the thinner fibers, indicating that they consist of similar actin/myosin complexes. The average diameters measured for the actin/myosin complies on the flat and fibrillar surfaces on days 1 and 4 are compared in Fig. 6C.

**Fig 7:**
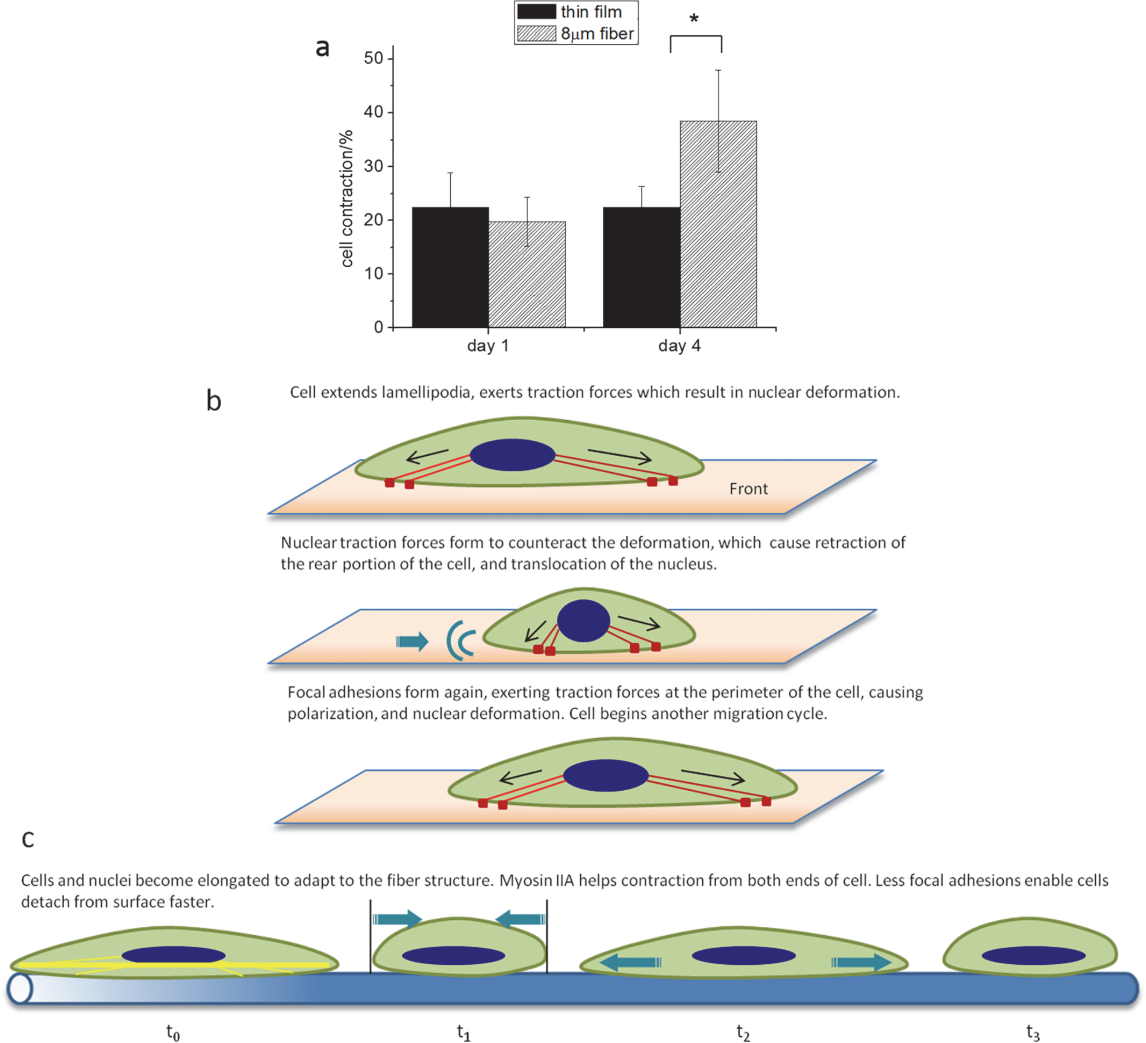
Cell contraction associated with migration on thin film and 8μm fiber—(a) Measurement of cell contraction on micron fibers and flat films at day 1 and day 4. Cell contraction on micron fiber is defined as: (cell length at t_0_- cell length at t_1_)/cell length at t_1_ *: P<0.001. (b) Illustration of cell contraction when migration on flat film. (c) Illustration of cell contraction when migration on 8μm fibers at day 4.

It has been demonstrated by numerous groups [32–34] that actin/myosin fibers are responsible for the exertion of traction forces which in turn mediate the process of cell contraction and translocation of the cell center of mass. We therefore measured the average amount of cell contraction on day 1 associated with migration and the degree of contraction associated with the migration on day 4 (Fig. 7A). From the figure we can see that the degree of contraction on day 1 on the fibrillar surface resembles that on the flat surface, but by the fourth day the degree of contraction on the fibers had nearly doubled, while the one on the flat surfaces remains unchanged.

Examination of the moving videos of the cells taken on days 1 and 4 (S1–S4 Movies) shows that this difference in contraction is a result of a complex set of motions which the cells undergo and which ultimately result in nuclear translocation or cell migration. In Fig. 7B and 7C, we illustrate the processes that are observed in the videos of the migrating cells. The motion on the flat surface is illustrated in Fig. 7B, where we observed a “fluttering” of the cytoplasm as the cell extends processes in multiple directions. Then as discussed in previous paper [18] we find that the nuclear shape changes abruptly, becoming more symmetric, which then causes retraction of the rear of the cell and nuclear translocation. Initially similar type of motion is observed on the fibrillar surface. On this surface one sees that most cells have one side attached to the sides of the fibers, while the other side is fluctuating freely. Initially, (first 24 hours) cell migration is similar on both types of surfaces but with increasing time the cells on the electrospun fibers appear to switch to a different mode. Instead of putting out processes in random directions, they are committed to following the direction of the fiber, and fluctuations of the periphery of the cytoplasm are no longer apparent. Hence the appearance of the large diameter of actin/myosin fibers also coincides with the increase in cell contraction and the rapid highly oriented motion.

## Discussion

Cell migration on fibrillar substrates appears to differ in a fundamental fashion from migration on planar surfaces. Since cells rarely migrate on flat planes in vivo, it is important to understand the changes induced on fibrillar surfaces. Two types of surfaces were prepared for these experiments, a flat spun cast PMMA film and 8μ diameter electrospun fibers. Both types of surfaces are not particularly cell adhesive, and therefore they were coated with fibronectin for 2 hours prior to plating the cells or the cell laden droplets. Hence the surfaces had identical chemical composition, but differed mostly in topography. On the flat surface the cells emerged en-mass from the droplet in a star burst trajectory with a velocity that decreased over the next 24 hours to the single cell value, as the cells separated. This value remained unchanged over the next seven days. For droplets placed on the fibrillar surfaces, the cells emerged only along the fibers. Careful examination indicated that most of the cells were attached on one side to the edge of the fibers with the other side of the cell on the adjacent flat surface. Immunohistochemical staining of vinculin proteins showed that on the fiber surfaces, the majority of the focal adhesions were concentrated in a straight line along the contact area with the fiber and along the periphery of the cell adherent to the flat surface. Very few focal adhesions were detected in the center of the cell. On the flat surfaces, on the other hand, even though some focal adhesions were seen along the leading and trailing edges, focal adhesions were distributed throughout the cell’s plasma membrane. As a result cells on flat surfaces had approximately 40% more focal adhesions after 24 hours. On the flat surfaces the number remained constant, while on the fibrillar surfaces they decreased by approximately 30% on day 4.

Focal adhesions are also responsible for anchoring the actin fibers and transmission of traction forces across the cytoplasm. Pan et al. [18] had shown that the distribution of the traction forces was also responsible for causing nuclear deformation, and initiating cellular translocation. Hence we postulated that the differences in focal adhesion distribution would also results in differences in the traction forces exerted and hence migration velocities and modes of cellular locomotion. Measurements of the cell migration velocity indeed showed that while the migration velocity remained constant on the flat surfaces, the velocity increased, nearly doubling, over the next four days for the cells on the fibrillar surface. This increase mirrored the decrease in focal adhesions, possibly enabling the faster motion. Another interesting observation though was the difference in nuclear deformation. The mean deformation on the fibrillar surfaces was nearly twice as large as on the flat surfaces, which was consistent with previous reports where cells were confined in porous substrates and migration was interrupted when the degree of confinement prevented nuclear deformation [18]. Furthermore, the nuclear deformation increased between days one and four, also mirroring the increase in cell migration velocity.

Observation of the mode of migration also showed differences between the flat and the fibrillar surfaces, where the differences were initially small on day 1, but increased and became very noticeable by day 4. On the flat surfaces migration was accompanied by a series of fluid deformations of the cytoplasm due to extension of lamellipodia, nuclear deformation and retraction of the rear of the cell, as shown in Fig. 7B. This mechanism was also observed initially on the fibers, on the first day, when the magnitude of the speed for cells migrating on both surfaces was similar. As the cells accelerated over the next three days on the fibrillar surfaces a different mode of migration started to spread. The cytoplasm contracted and expanded, in a manner similar to muscle cells, while the nucleus remained highly deformed. While RT-PCR and immunofluorescence indicated that the amount of myosin did not change for the cells on the fibrillar surface during the four day observation period, immunofluorescence images showed that large myosin/actin fibers had formed. Measurements of the cell contraction also showed that during this time the contraction amplitude doubled for cell migrating on the fibers, while remaining unchanged for those migrating on the flat surface, suggesting that these larger fibers, were more efficient at contracting the cells.

Taken together, these results indicate that the continual deformation of the cells imposed by the fiber topography was responsible for triggering a different mode of migration, which is more oriented, faster, and more efficient than that observed on flat surfaces.

## Supporting Information

S1 Movie Cell migration on fibers at day 1. (MP4)

S2 Movie Cell migration on fibers at day 4. (MP4)

S3 Movie Cell migration on thin film at day 1. (MP4)

S4 Movie Cell migration on thin film at day 4. (MP4)
